# Induction of long intergenic non-coding RNA HOTAIR in lung cancer cells by type I collagen

**DOI:** 10.1186/1756-8722-6-35

**Published:** 2013-05-13

**Authors:** Yan Zhuang, Xiang Wang, Hong T Nguyen, Ying Zhuo, Xinpeng Cui, Claire Fewell, Erik K Flemington, Bin Shan

**Affiliations:** 1 Department of Medicine and Pathology, Tulane University School of Medicine, 1430 Tulane Ave. SL-9, New Orleans, LA, 70112, USA; 2 Department of Thoracic Surgery, The Second Xiangya Hospital of Central South University, Changsha, Hunan, 410078, P.R. China; 3 Department of Medicine and Pathology, Tulane University School of Medicine, 1430 Tulane Ave. SL-9, New Orleans, LA 70112, USA

**Keywords:** HOTAIR, lincRNA, Type I collagen, Three-dimensional ogranotypic culture

## Abstract

**Background:**

The tumor microenvironment is a crucial determinant in tumor progression. Interstitial extracellular matrix (ECM), such as type I collagen (Col-1), is aberrantly enriched in the tumor microenvironment and promotes tumor progression. Long intergenic non-coding RNAs (lincRNA) are a new family of regulatory RNAs that modulate fundamental cellular processes via diverse mechanisms.

**Findings:**

We investigated whether the expression of lincRNAs was regulated by the tumor promoting Col-1. In a three-dimensional organotypic culture model using the reconstituted basement membrane ECM Matrigel (rBM 3-D), supplementation of Col-1 disrupted acini, a differentiation feature of well-differentiated lung adenocarcinoma cells, and concurrently induced the expression of a tumor-promoting lincRNA, HOX transcript antisense RNA (HOTAIR). Induction of HOTAIR by Col-1 was diminished by a neutralizing antibody against the Col-1 receptor α2β1 integrin. Col-1 activates the expression of a reporter gene controlled by the human HOTAIR promoter. Moreover the expression of HOTAIR and Col-1 was concurrently up-regulated in human non-small cell lung cancer.

**Conclusions:**

Our findings indicate that tumor-promoting Col-1 up-regulates the expression of HOTAIR in NSCLC cells. These initial results warrant further investigation of HOTAIR and other lincRNA genes in lung tumorigenesis.

## Findings

The tumor microenvironment is aberrantly enriched with interstitial extracellular matrix (ECM), such as type I collagen (Col-1) [1]. The tumor-promoting activity of Col-1 has been successfully investigated using three-dimensional organotypic culture based on reconstituted basement membrane matrix Matrigel (rBM 3-D) [2]. In rBM 3-D culture, normal lung and mammary epithelial cells form acini, a differentiation feature that manifests spheres of polarized epithelial cells enclosing a central lumen [3,4]. Col-1 disrupts acinar morphogenesis of lung and mammary epithelial cells in rBM 3-D culture and promotes tumor progression *in vivo*[5,6]. Moreover, Col-1 up-regulates the expression of several oncogenic miRNAs, such as miR-21 in rBM 3-D culture of lung and mammary epithelial cells [6,7].

Long intergenic non-coding RNAs (lincRNAs) are a new family of regulatory RNAs that modulate tumorigenesis via diverse mechanisms [8]. HOX transcript antisense RNA (HOTAIR) is a HOXC cluster-derived lincRNA that binds to the transcriptional co-repressor polycomb repressive complex 2 (PRC2) and recruits PRC2 to silence its target genes [9]. HOTAIR is proposed as an oncogene because its expression is elevated in several types of cancers and mediates invasion and metastasis of breast cancer cells [10-12]. Regulation of the expression of HOTAIR in cancer remains unclear and is investigated in the current study.

**Figure 1:**
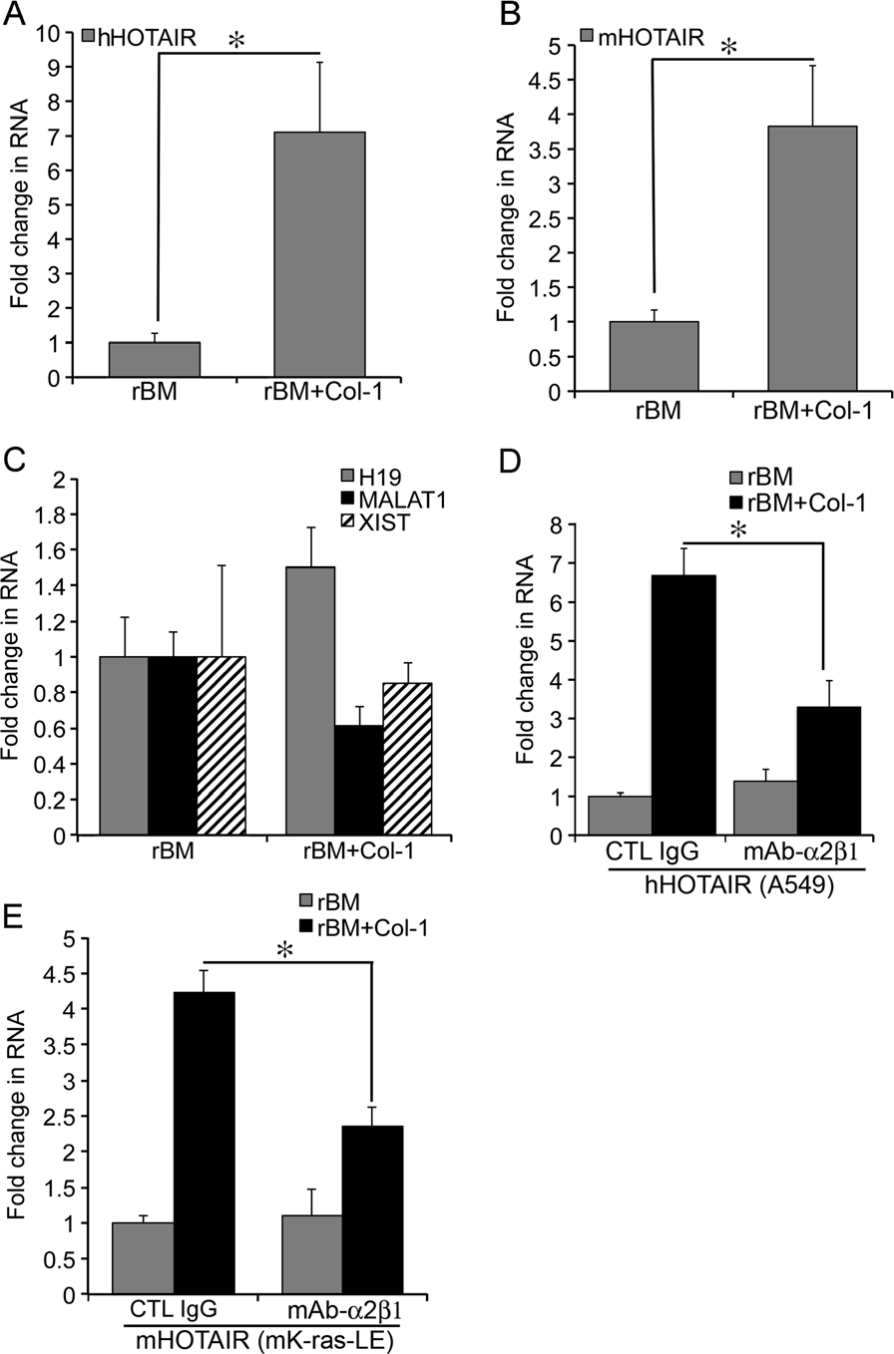
**Induction of HOTAIR Expression by Col-1. A**) Total cell RNA was extracted from rBM 3-D ± Col-1 cultures of A549 cells. The RNA levels of HOTAIR were measured using qRT-PCR and normalized to the house-keeping gene 36B4. A fold change was obtained setting the values from rBM 3-D culture to one. **B**) Similar to part A except that the RNA levels of mouse HOTAIR were compared between rBM ± Col-1 3-D cultures of mK-ras-LE cells. **C**) Similar to part A except that the RNA levels of H19, MALAT1, and XIST were compared between rBM ± Col-1 3-D cultures of A549 cells. **D**) The RNA levels of HOTAIR were measured using qRT-PCR in rBM ± Col-1 3-D culture of A549 cells with or without an integrin α2β1 neutralizing antibody (mAb-α2β1). A fold change in the HOTAIR transcript was obtained by setting the values from the control IgG group (CTL IgG) to one. **E**) Similar to part D except that the experiments were carried out in mK-ras-LE cells. Mean and standard deviations were obtained from three independent experiments. * indicated a *P* value < 0.05 as determined using unpaired two tail student *T* test.

**Figure 2:**
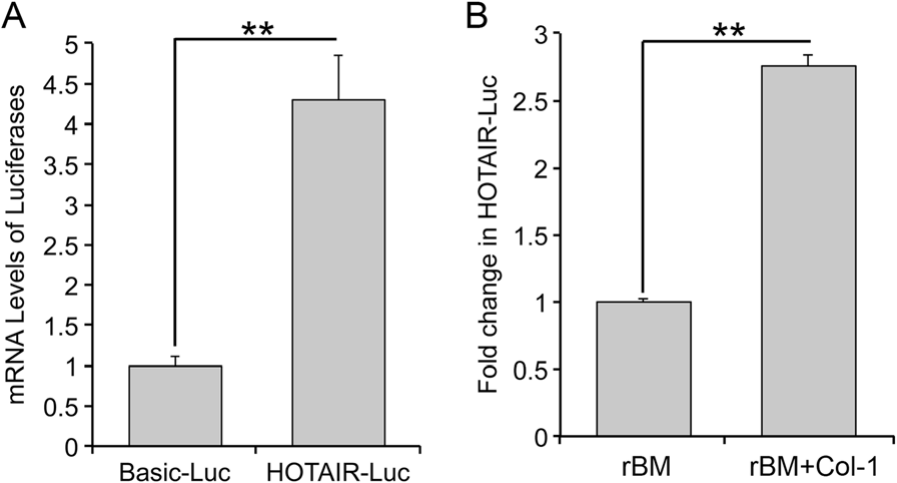
**Activation of the human HOTAIR promoter by Col-1. A**) A549 cells were transfected with either pGL3 Basic luciferase reporter (Basic-Luc) or the human HOTAIR promoter-controlled luciferase reporter in the pGL3 Basic backbone (HOTAIR-Luc). The mRNA levels of the firefly luciferase reporter were measured using qRT-PCR and normalized to the co-transfected renilla luciferase reporter. A fold change in the expression of the firefly luciferase reporter was obtained by setting the values of Basic-Luc to one. **B**) The mRNA levels of the HOTAIR promoter-controlled luciferase reporter were measured in rBM ± Col-1 3-D cultures of A549 cells using qRT-PCR. A fold change in the mRNA levels of the luciferase reporter was obtained by setting the values from the rBM 3-D culture to one. Mean and standard deviations were obtained from three independent experiments. ** indicated a *P* value < 0.01 as determined using unpaired two tail student *T* test.

Induction of HOTAIR Expression by Type I Collagen — Because of the tumor-promoting activities of Col-1 and HOTAIR, we sought to determine whether Col-1 regulated the expression of HOTAIR during disruption of acinar morphogenesis of lung epithelial cells. A549 cells, a human lung adenocarcinoma cell line, and mK-ras-LE cells, a murine lung adenocarcinoma cell line, undergo acinar morphogenesis in Matrigel (BD Biosciences, Rockville MD) based rBM 3-D culture [4,6,13-15]. As revealed by phase contrast and confocal fluorescent microscopy A549 and mK-ras-LE cells grow as polarized monolayer cell spheres enclosing a central lumen [16]. We compared the RNA levels of HOTAIR between the rBM 3-D ± Col-1 cultures using qRT-PCR (primer sequences listed Additional file 1: Table S1) [17]. We supplemented rBM 3-D culture with 2 mg/ml of Col-1 because this concentration of Col-1 in rBM 3-D culture yields a rigidity that is comparable to that observed in the stiff tumor microenvironment of patients with breast cancer [5,18,19]. Addition of Col-1 (Sigma, St. Louis MO) into rBM 3-D culture induced a 7.1- and 3.8-fold increase in the HOTAIR RNA levels in A549 and mK-ras-LE cells, respectively (Figure 1A & B). On the other hand Col-1 did not significantly alter the expression of three other lincRNA genes, namely H19, XIST, or MALAT1 in A549 cells (Figure 1C). Because integrin α2β1 is the cell surface receptor of Col-1, we inhibited Col-1 signaling using an integrin α2β1 neutralizing antibody (clone JBS2, Chemicon, Temecula CA) [20]. The integrin α2β1 neutralizing antibody (10 μg/ml) reduced the Col-1-induced HOTAIR to 50% and 55% of that in the control IgG treated A549 cells and mK-ras-LE cells, respectively (Figure 1D & E). These results further confirmed Col-1-mediated expression of HOTAIR. To determine whether the promoter activity of the human HOTAIR gene was regulated by Col-1, we inserted 1 kb DNA fragment of the human HOTAIR gene immediate upstream from its transcription start site into the pGL3 Basic luciferase reporter (HOTAIR-Luc whose construction was described in details in the Additional file 1: Figure S1) (Promega, Madison WI). Because of technical limitation of using passive lysis buffer to extract Luc from rBM 3-D culture, we measured the expression of Luc reporter using qRT-PCR as we previously described [21]. To confirm transcriptional regulatory activity of the HOTAIR promoter insert, we compared Luc expression controlled by the HOTAIR promoter (HOTAIR-Luc) and by the minimal promoter in the backbone vector pGL3 Basic (Basic-Luc). HOTAIR-Luc exhibited a 4.3-fold increase in Luc expression over that of Basic-Luc (Figure 2A, *P* = 0.004 as determined using unpaired two tail student *T* test). The data confirmed the transcriptional activity of the cloned HOTAIR promoter in A549 cells. We then compared the expression of HOTAIR-Luc between rBM 3-D cultures of A549 cells with or without Col-1. A549 cells were transfected with the reporter plasmids using Lipofectamine 2000 in a reverse fashion [22,23]. The transfected cells were immediately seeded into rBM 3-D culture after transfection. Consistent with its induction of the endogenous HOTAIR gene, Col-1 induced a 2.7-fold increase in HOTAIR-Luc activity over that in the control rBM 3-D culture (Figure 2B, Figure 2A, *P* = 0.002 as determined using unpaired two tail student *T* test). These findings suggest that Col-1 transcriptionally activates the expression of HOTAIR.

**Figure 3:**
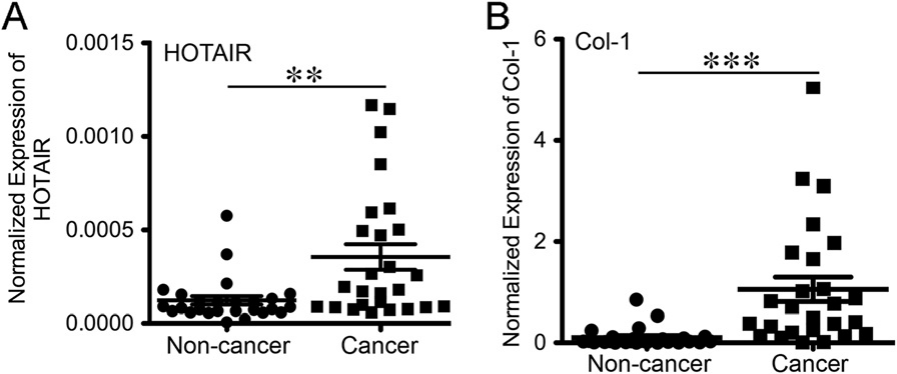
**Elevated expression of HOTAIR and Col-1 in human NSCLC. A**) The RNA levels of HOTAIR were measured using qRT-PCR in the RNA samples extracted from the cancer and non-cancer tissue specimens of the patients with NSCLC. The RNA levels of HOTAIR were measured using qRT-PCR and normalized to the house keeping gene β-actin. **B**) Similar to part A except that the mRNA levels of Col-1 were compared between cancer and non-cancer tissues. Means and standard deviations were obtained from 26 pairs of cancer and non-cancer specimens. ** and *** indicated a *P* value < 0.01 and 0.001 respectively as determined by paired two tail student *T* test.

Elevated Expression HOTAIR in NSCLC — We then questioned whether the expression of HOTAIR was elevated in human non-small cell lung cancer (NSCLC). The testing group consisted of 26 pairs of fresh cancer and non-cancer tissues that were collected during resection of the primary tumors from the patients with non-small cell lung cancer (NSCLC) at the Second Xiangya Hospital of Central South University from 2010 to 2011. The detailed clinical characteristics of the enrolled patients (gender, age, smoking history, and TNM staging) were provided in Additional file 1: Table S2. None of the patients received any pre-operative therapies. The protocol for collection of the specimens and the related clinical data abided by the principles laid down in the Helsinki Declaration and was approved by the Ethical Committee of the Second Xiangya Hospital. In 26 pairs of cancer and matching non-cancer tissues from patients with NSCLC, the RNA levels of HOTAIR in cancer tissues exhibited a 4.8-fold increase over that in the corresponding non-cancer tissues (*P* = 0.002 as determined using paired two tail student *T* test) (Figure 3A). Concurrently the mRNA levels of Col-1 in cancer tissues exhibited a 16.8-fold increase over that in the corresponding non-cancer tissues (*P* = 0.0003 as determined using paired two tail student *T* test) (Figure 3A). Despite overall upward trend of HOTAIR and Col-1 expression in cancer tissues, we did not observe a quantitative correlation between HOTAIR and Col-1. Col-1 exhibited a broader and greater increase in the group (>1.5-fold in 25 patients) than HOTAIR (>1.5-fold in 14 patients) (Additional file 1: Table S2).

In the current study, we demonstrate that the tumor-promoting lincRNA HOTAIR is induced by Col-1 and its expression inversely correlates with acinar morphogenesis, a differentiation feature of lung epithelial cells in rBM 3-D culture (Figure 1) [4]. These *in vitro* findings suggest that the elevated HOTAIR expression in tumor tissues results from cancer cells’ response to Col-1 that is aberrantly enriched in the tumor microenvironment (Figure 3) [2,5,19]. Col-1 appears to transcriptionally activate the HOTAIR gene because Col-1 activates a reporter gene controlled by the human HOTAIR promoter (Figure 2). In silico analysis of the human HOTAIR promoter using the geWorkbench Promoter Scanning Module reveals four potential Myc binding sites (https://wiki.nci.nih.gov/display/geWorkbench/geWorkbench) (Additional file 1: Table S3). It is conceivable that Myc mediates activation of the HOTAIR gene by Col-1 because the miR-17-92 cluster, another transcriptional target of Myc in cancer cells, is concurrently up-regulated by Col-1 in rBM 3-D culture in our recent report [6,24]. The clinical relevance of our findings is supported by a concurrent up-regulation of HOTAIR and Col-1 expression in NSCLC (Figure 3). A lack of quantitative correlation between HOTAIR and Col-1 expression implicates additional signaling in regulation of the expression of HOTAIR in NSCLC. Because the specimens were collected recently, it is unclear whether the elevated expression of HOTAIR holds prognostic values in NSCLC as in several other types of tumors [10-12]. The patients are enrolled in a follow-up study to monitor progression and survival.

To the best of our knowledge, the current study is the very first attempt to examine a link between the tumor microenvironment and lincRNAs using rBM 3-D organotypic culture. Because HOTAIR can epigenetically reprogram global chromatin staging and gene expression via its interaction with PRC2, our initial findings warrant further investigation of HOTAIR-mediated epigenetic regulation of the crosstalk between cancer cells and their microenvironment.

## Abbreviations

ECM: Extracellular matrix; Col-1: Type I collagen; lincRNA: Long intergenic non-coding RNA; HOTAIR: HOX transcript antisense RNA; rBM 3-D: Reconstituted basement membrane matrix Matrigel based three-dimensional culture; PRC2: Polycomb repressive complex 2; NSCLC: Non-small cell lung cancer.

## Competing interests

The authors declare that they have no competing interests.

## Authors’ contribution

YZ and HTN contributed to data collection and analysis of rBM 3-D culture. XW, and XC contributed to data collection and analysis of patient specimens. CF and EKF contributed to the analysis of the human HOTAIR promoter. BS conceived the study and wrote the manuscript. All authors read and approved the final manuscript.

## Supplementary Material

Additional file 1: Table S1 Sequences of the primers (human HOTAIR: NR_003716; mouse HOTAIR: NR_047528). **Table S2.** Clinical characteristics of the recruited patients. **Table S3.** Scanning of the human HOTAIR promoter. **Figure S1.** Construction of HOTAIR-Luc. Click here for file
