# Supplementary material for: Induction of long intergenic non-coding RNA HOTAIR in lung cancer cells by type I collagen
Source: J Hematol Oncol. 2013 May 13;6:35. doi: 10.1186/1756-8722-6-35 (PMC3655931; doi:10.1186/1756-8722-6-35)
Supplement: Additional file 1: Table S1 — Sequences of the primers (human HOTAIR: NR_003716; mouse HOTAIR: NR_047528). Table S2. Clinical characteristics of the recruited patients. Table S3. Scanning of the human HOTAIR promoter. Figure S1. Construction of HOTAIR-Luc. [file 1756-8722-6-35-S1.pdf]

Supplementary Materials Table 1. Sequences of the primers (human HOTAIR: NR_003716; mouse HOTAIR: NR_047528)

hHOTAIR-F	5'-AAATATGGCGGCGTCTACACGGAA-3'
hHOTAIR-R	5'-TCCAGAACCCTCTGACATTTGCCT-3'
mHOTAIR-F	5'-TCCAGATGGAAGGAACTCCAGACA-3'
mHOTAIR-R	5'-ATAGATGTGCGTGGTCAGATCGCT-3'

Supplementary Materials Table 2. Clinical characteristics of the recruited patients. A fold change of HOTAIR and Col-1 was presented as ratios of the expression in cancer tissues over their corresponding non cancer tissues. M: male; F: female; Adeno: adenocarcinoma; Squamous: squamous carcinoma; Y: yes; N: no; CA: cancer; NC: non cancer.

ID #	Age	Gender	Smoking history	Pathology	TNM staging	HOTAIR (CA/NC)	Col-1 (CA/NC)
1	47	M	Y	Adeno	T2N1M0	0.6	26.9
2	55	F	N	Adeno	T1N2M0	0.8	2.2
3	61	M	N	Adeno	T2N1M0	0.8	2.9
4	62	M	N	Adeno	T2N2M0	0.9	8.5
5	47	M	Y	Squamous	T2N1M0	0.9	3.7
6	73	F	N	Squamous	T1N0M0	0.9	14.9
7	59	M	N	Squamous	T2N0M0	1.2	10.8
8	61	M	Y	Adeno	T1N0M0	1.3	22.9
9	48	M	Y	Adeno	T1N0M0	1.3	36.5
10	55	M	Y	Adeno	T1N2M0	1.3	6.5
11	63	F	N	Adeno	T2N1M0	1.4	1.9
12	56	M	N	Adeno	T2N2M0	1.4	10.4
13	49	F	N	Squamous	T2N0M0	1.5	38.3
14	62	M	N	Squamous	T2N0M0	1.6	2.4
15	60	M	Y	Squamous	T2N0M0	2.1	8.1
16	62	F	N	Adeno	T2N0M0	2.2	106.9
17	51	M	Y	Squamous	T2N0M0	3.1	0.9
18	71	M	Y	Squamous	T2N0M0	3.4	3.5
19	47	M	Y	Adeno	T2N1M0	3.5	58.0
20	56	F	N	Adeno	T1N2M0	6.7	6.4
21	38	M	Y	Adeno	T2N2M0	9.5	4.4
22	54	F	N	Adeno	T1N0M0	10.5	17.2
23	78	F	N	Squamous	T2N0M0	10.7	6.7
24	60	M	N	Adeno	T2N2M0	11.9	15.8
25	62	M	N	Adeno	T2N2M0	17.4	7.6
26	44	M	Y	Squamous	T2N0M0	28.4	11.5

Supplementary Materials Table 3. Scanning of the human HOTAIR promoter.

Trasnscription Factor	Start	End
AP1:Leucine Zipper:MA0099	881	887
Ar:Hormone-nuclear Receptor:MA0007	229	208
ELK4:Ets:MA0076	881	873
En1:Homeo:MA0027	757	747
ESR2:Hormone-nuclear Receptor:MA0258	734	717
Evi1:BetaBetaAlpha-zinc finger:MA0029	837	850
FEV:Ets:MA0156	502	509
Foxd3:Forkhead:MA0041	925	914
Foxd3:Forkhead:MA0041	929	918
Foxd3:Forkhead:MA0041	949	960
FOXF2:Forkhead:MA0030	169	156
FOXF2:Forkhead:MA0030	905	918
FOXF2:Forkhead:MA0030	911	924
FOXI1:Forkhead:MA0042	818	807
FOXL1:Forkhead:MA0033	90	83
FOXL1:Forkhead:MA0033	439	432
Foxq1:Forkhead:MA0040	762	772
Foxq1:Forkhead:MA0040	818	808
GABPA:Ets:MA0062	228	218
GABPA:Ets:MA0062	393	383
Gata1:GATA:MA0035	168	158
GATA3:GATA:MA0037	401	406
Gfi:BetaBetaAlpha-zinc finger:MA0038	407	398
Hand1::Tcfe2a:Helix-Loop-Helix:MA0092	571	562
Hand1::Tcfe2a:Helix-Loop-Helix:MA0092	907	898
HLF:Leucine Zipper:MA0043	879	890
Hltf:GATA:MA0109	587	596
INSM1:BetaBetaAlpha-zinc finger:MA0155	194	183
INSM1:BetaBetaAlpha-zinc finger:MA0155	947	936
IRF1:IRF:MA0050	77	66
IRF1:IRF:MA0050	559	548
IRF1:IRF:MA0050	886	897
IRF2:IRF:MA0051	78	61
IRF2:IRF:MA0051	478	461
IRF2:IRF:MA0051	560	543
IRF2:IRF:MA0051	832	849
IRF2:IRF:MA0051	909	926
Klf4:BetaBetaAlpha-zinc finger:MA0039	194	185
Klf4:BetaBetaAlpha-zinc finger:MA0039	385	376
Klf4:BetaBetaAlpha-zinc finger:MA0039	596	605
Lhx3:Homeo:MA0135	827	815
Mafb:Leucine Zipper:MA0117	761	754

MEF2A:MADS:MA0052	452	461
MEF2A:MADS:MA0052	690	699
MIZF:BetaBetaAlpha-zinc finger:MA0131	711	702
MIZF:BetaBetaAlpha-zinc finger:MA0131	752	761
Myb:Myb:MA0100	564	571
**MYC::MAX:Helix-Loop-Helix:MA0059**	**494**	**484**
**MYC::MAX:Helix-Loop-Helix:MA0059**	**728**	**738**
**Mycn:Helix-Loop-Helix:MA0104**	**4**	**13**
**Mycn:Helix-Loop-Helix:MA0104**	**729**	**738**
MZF1_1-4:BetaBetaAlpha-zinc finger:MA0056	277	272
MZF1_1-4:BetaBetaAlpha-zinc finger:MA0056	340	345
NF-kappaB:Rel:MA0061	183	174
NF-kappaB:Rel:MA0061	601	610
NFATC2:Rel:MA0152	837	831
NKX3-1:Homeo:MA0124	135	129
NR1H2::RXRA:Hormone-nuclear Receptor:MA0115	264	248
NR3C1:Hormone-nuclear Receptor:MA0113	596	579
Pax2:Homeo:MA0067	106	113
Pax4:Homeo:MA0068	876	847
Pax4:Homeo:MA0068	877	848
Pax4:Homeo:MA0068	912	941
Pax4:Homeo:MA0068	913	942
PBX1:Homeo:MA0070	818	829
Pdx1:Homeo:MA0132	59	54
PLAG1:BetaBetaAlpha-zinc finger:MA0163	371	358
PLAG1:BetaBetaAlpha-zinc finger:MA0163	381	368
PPARG::RXRA:Hormone-nuclear Receptor:MA0065	264	250
PPARG:Hormone-nuclear Receptor:MA0066	328	309
REL:Rel:MA0101	140	149
REST:BetaBetaAlpha-zinc finger:MA0138	164	184
RORA_1:Hormone-nuclear Receptor:MA0071	146	155
RREB1:BetaBetaAlpha-zinc finger:MA0073	367	386
RREB1:BetaBetaAlpha-zinc finger:MA0073	907	926
RUNX1:Runt:MA0002	152	162
RUNX1:Runt:MA0002	646	636
RXRA::VDR:Hormone-nuclear Receptor:MA0074	864	878
SOX10:High Mobility Group:MA0442	890	885
SPIB:Ets:MA0081	394	388
SRF:MADS:MA0083	439	450
SRY:High Mobility Group:MA0084	910	918
SRY:High Mobility Group:MA0084	916	924
STAT1:Stat:MA0137	435	449
TBP:TATA-binding:MA0108	441	455

TBP:TATA-binding:MA0108	748	734
Tcfcp2l1 :CP2:MA0145	182	195
USF1:Helix-Loop-Helix:MA0093	11	5
USF1:Helix-Loop-Helix:MA0093	6	12
ZEB1:BetaBetaAlpha-zinc finger:MA0103	882	877

Supplementary Materials Figure 1. Construction of HOTAIR-Luc

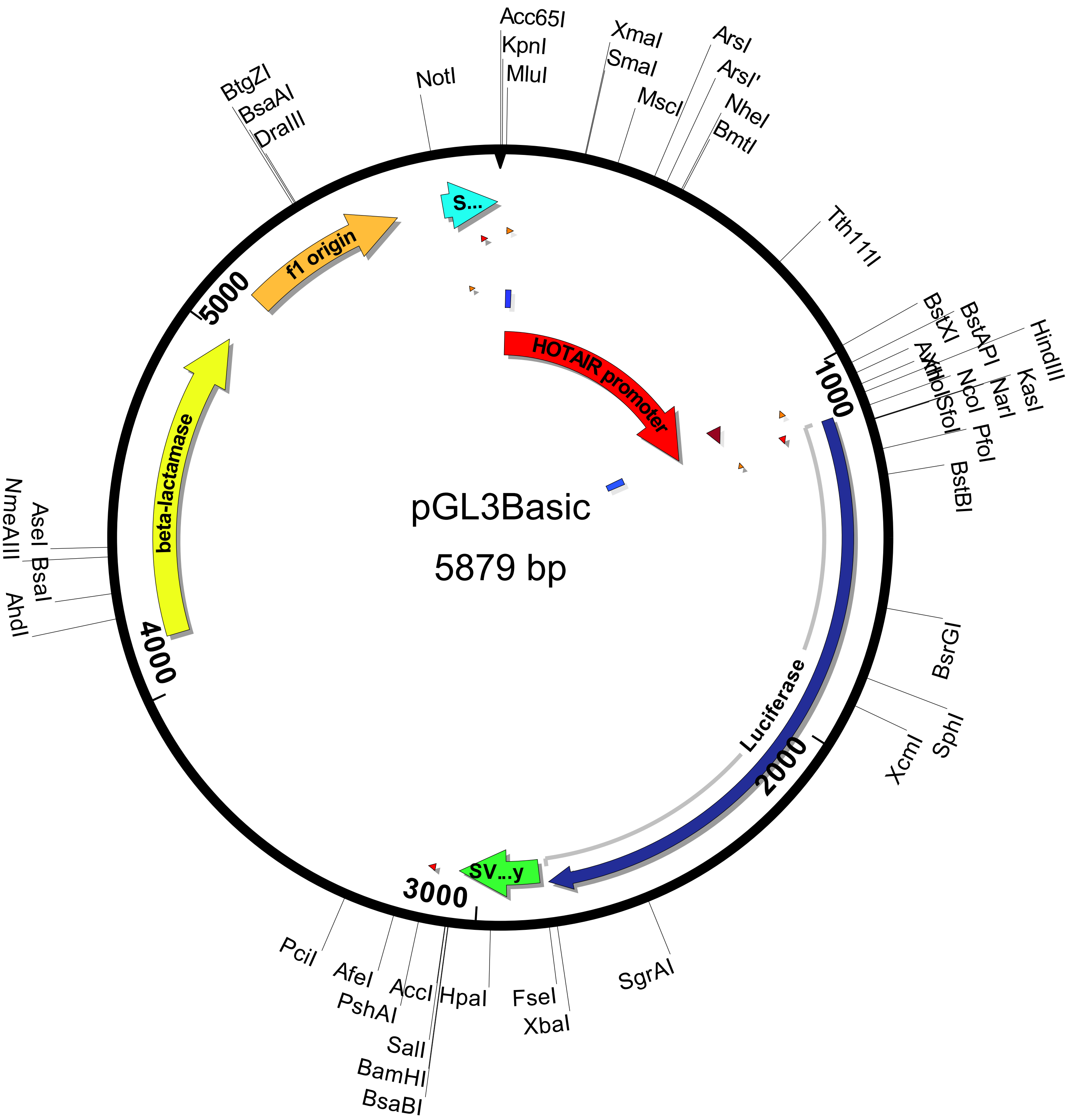

Acc65I KpnI MluI

5' GGTACCGAGCTCTTACGCGTTAAGCGGAGAGAGTCCCACACATGGACGCTCTCGTTTGTTCACAGGCTGGGCCAGCAAGGAGACTTCATTAGAAATTGGCTTTCACCTTC 110
3' CCATGGCTCGAGAATGCGCAATTCGCCTCTCTCAGGGTGTGTACCTGCGAGAGCAAACAAGTGTCCGACCCGGTCGTTCCTCTGAAGTAATCTTTAACCGAAAGTGGAAG

HOTAIR MluI 626 fwd

5' Primer region

HOTAIR promoter

XmaI SmaI

5' TTGCCTATATTTCTCTCCCTTACAGTAATATCACTGCCTGCAATAATCCCTTTAGTATGCTTTGGGATTTTCAGGTCAGTGGTTTATCTTGCACCCGGGACACCCCAGCC 220
3' AACGGATATAAAGAGAGGGAATGTCATTATAGTGACGGACGTTATTAGGGAAATCATACGAAACCCTAAAAGTCCAGTCACCAAATAGAACGTGGGCCCTGTGGGGTCGG

HOTAIR promoter

MscI

5' CCAGCCAGGGAACAAGAAGCGGGTGGGACTGCTAGTTCCTGGCTTCCACCTCTTCTCAGACTTGGCCATTGCCCCAAGCCTGAGTCCCCAAGTCAGGCCACCTAAGCTGG 330
3' GGTCGGTCCCTTGTTCTTCGCCCACCCTGACGATCAAGGACCGAAGGTGGAGAAGAGTCTGAACCGGTAACGGGGTTCGGACTCAGGGGTTCAGTCCGGTGGATTCGACC

HOTAIR promoter

ArsI ArsI'

5' TGGACGCTGCTCAGAGGTTCCTGGGACCTATGGAGGAGGTAGTGGGGAGGGAGAAGGACAGCTCCTTTGCCCCCACAGGCCCCACCCAGCTTCCTCTGTGGAGAGATTGT 440
3' ACCTGCGACGAGTCTCCAAGGACCCTGGATACCTCCTCCATCACCCCTCCCTCTTCCTGTCGAGGAAACGGGGGTGTCCGGGGTGGGTCGAAGGAGACACCTCTCTAACA

HOTAIR promoter

NheI BmtI

5' CCCAGGATGCTAGCAACCCGACCCTATTTCTTCCCATAAAAGCCCTATTTTACGAGCCGGTTTCACTGGCCCCCTCACCAAATGCTCCAAAATGAAGGAAATGTCTTAAA 550
3' GGGTCCTACGATCGTTGGGCTGGGATAAAGAAGGGTATTTTCGGGATAAAATGCTCGGCCAAAGTGACCGGGGGAGTGGTTTACGAGGTTTTACTTCCTTTACAGAATTT

HOTAIR promoter

5' AGCAGGCTGGAAATGGAGCCGCTGCTATTGGGCTTCAGTTTCCTAAGCCAGTTGTAGTGGCACAGAAAATACCTTTTCTGGGTGGGATTGCCCTGTTGGGCCACCCAGAT 660
3' TCGTCCGACCTTTACCTCGGCGACGATAACCCGAAGTCAAAGGATTCGGTCAACATCACCGTGTCTTTTATGGAAAAGACCCACCCTAACGGGACAACCCGGTGGGTCTA

HOTAIR promoter

Tth111I

5' CTCCCGCCTCACCACAGAGCAGAGGCGGGTTGGCCGGAGCCCTGATTCTCTGGCTTTAGCTCCTACATTAAGGCTCGGACCTAGTCCTCCTGATCAAAGTGAGCTCGCGG 770
3' GAGGGCGGAGTGGTGTCTCGTCTCCGCCCAACCGGCCTCGGGACTAAGAGACCGAAATCGAGGATGTAATTCCGAGCCTGGATCAGGAGGACTAGTTTCACTCGAGCGCC

HOTAIR promoter

5' CATTTTTATGAGATCAACTTCAGCAACTCTTTATTAGCGATCAGATGCCTGGGTCGAAAATGCAAAAGGGAACAAACAGCTAATCAATCCAAAAGGAAAAAGAAAAGAGA 880
3' GTAAAAATACTCTAGTTGAAGTCGTTGAGAAATAATCGCTAGTCTACGGACCCAGCTTTTACGTTTTCCCTTGTTTGTCGATTAGTTAGGTTTTCCTTTTTCTTTTCTCT

HOTAIR promoter

5' GGGGTGGGAAGGCATGGGGTGAAAAACTTCAGGTGACACAAAGCAAAAAGATTCCAGTCACCAAAAACAAAAACAAAAAAAAAcCTTTTGCCCCCAGCAAGAAcCATTTG 990
3' CCCCACCCTTCCGTACCCCACTTTTTGAAGTCCACTGTGTTTCGTTTTTCTAAGGTCAGTGGTTTTTGTTTTTGTTTTTTTTTgGAAAACGGGGGTCGTTCTTgGTAAAC

HOTAIR promoter

BstXI BstAPI AvrII XhoI

5' TTTGGGATCTGTTCCAGCCTCCAGGCCCTGCCTTCTGCCTGCACATTCTGCCCTGATTTCCGGAACCTGGAAGCCTAGGCAGGCAGTGGGGAACTCTGACTCCTCGAGAT 1100

3' AAACCCTAGACAAGGTCGGAGGTCCGGGACGGAAGACGGACGTGTAAGACGGGACTAAAGGCCTTGGACCTTCGGATCCGTCCGTCACCCCTTGAGACTGAGGAGCTCTA

HOTAIR promoter

HOTAIR XhoI 1697 rev

Exon 1

3' Primer region

KasI NarI SfoI
HindIII NcoI

5' CTGCGATCTAAGTAAGCTTGGCATTCCGGTACTGTTGGTAAAGCCACCATGGAAGACGCCAAAAACATAAAGAAAGGCCCGGCGCCATTCTATCCGCTGGAAGATGGAAC 1210

3' GACGCTAGATTCATTCGAACCGTAAGGCCATGACAACCATTTCGGTGGTACCTTCTGCGGTTTTTGTATTTCTTTCCGGGCCGCGGTAAGATAGGCGACCTTCTACCTTG

Luciferase

GLprimer2 binding site

pGL3 Basic reverse primer

PfoI BstBI

5' CGCTGGAGAGCAACTGCATAAGGCTATGAAGAGATACGCCCTGGTTCCTGGAACAATTGCTTTTACAGATGCACATATCGAGGTGGACATCACTTACGCTGAGTACTTCG 1320

3' GCGACCTCTCGTTGACGTATTCCGATACTTCTCTATGCGGGACCAAGGACCTTGTTAACGAAAATGTCTACGTGTATAGCTCCACCTGTAGTGAATGCGACTCATGAAGC

Luciferase

5' AAATGTCCGTTCGGTTGGCAGAAGCTATGAAACGATATGGGCTGAATACAAATCACAGAATCGTCGTATGCAGTGAAAACTCTCTTCAATTCTTTATGCCGGTGTTGGGC 1430

3' TTTACAGGCAAGCCAACCGTCTTCGATACTTTGCTATACCCGACTTATGTTTAGTGTCTTAGCAGCATACGTCACTTTTGAGAGAAGTTAAGAAATACGGCCACAACCCG

Luciferase

5' GCGTTATTTATCGGAGTTGCAGTTGCGCCCGCGAACGACATTTATAATGAACGTGAATTGCTCAACAGTATGGGCATTTCGCAGCCTACCGTGGTGTTCGTTTCCAAAAA 1540

3' CGCAATAAATAGCCTCAACGTCAACGCGGGCGCTTGCTGTAAATATTACTTGCACTTAACGAGTTGTCATACCCGTAAAGCGTCGGATGGCACCACAAGCAAAGGTTTTT

Luciferase

BsrGI

5' GGGGTTGCAAAAAATTTTGAACGTGCAAAAAAAGCTCCCAATCATCCAAAAAATTATTATCATGGATTCTAAAACGGATTACCAGGGATTTCAGTCGATGTACACGTTCG 1650

3' CCCCAACGTTTTTTAAAACTTGCACGTTTTTTTCGAGGGTTAGTAGGTTTTTTAATAATAGTACCTAAGATTTTGCCTAATGGTCCCTAAAGTCAGCTACATGTGCAAGC

Luciferase

5' TCACATCTCATCTACCTCCCGGTTTTAATGAATACGATTTTGTGCCAGAGTCCTTCGATAGGGACAAGACAATTGCACTGATCATGAACTCCTCTGGATCTACTGGTCTG 1760

3' AGTGTAGAGTAGATGGAGGGCCAAAATTACTTATGCTAAAACACGGTCTCAGGAAGCTATCCCTGTTCTGTTAACGTGACTAGTACTTGAGGAGACCTAGATGACCAGAC

Luciferase

SphI

5' CCTAAAGGTGTCGCTCTGCCTCATAGAACTGCCTGCGTGAGATTCTCGCATGCCAGAGATCCTATTTTTGGCAATCAAATCATTCCGGATACTGCGATTTTAAGTGTTGT 1870

3' GGATTTCCACAGCGAGACGGAGTATCTTGACGGACGCACTCTAAGAGCGTACGGTCTCTAGGATAAAAACCGTTAGTTTAGTAAGGCCTATGACGCTAAAATTCACAACA

Luciferase

XcmI

5' TCCATTCCATCACGGTTTTGGAATGTTTACTACACTCGGATATTTGATATGTGGATTTCGAGTCGTCTTAATGTATAGATTTGAAGAAGAGCTGTTTCTGAGGAGCCTTC 1980
3' AGGTAAGGTAGTGCCAAAACCTTACAAATGATGTGAGCCTATAAACTATACACCTAAAGCTCAGCAGAATTACATATCTAAACTTCTTCTCGACAAAGACTCCTCGGAAG

Luciferase

5' AGGATTACAAGATTCAAAGTGCGCTGCTGGTGCCAACCCTATTCTCCTTCTTCGCCAAAAGCACTCTGATTGACAAATACGATTTATCTAATTTACACGAAATTGCTTCT 2090
3' TCCTAATGTTCTAAGTTTCACGCGACGACCACGGTTGGGATAAGAGGAAGAAGCGGTTTTCGTGAGACTAACTGTTTATGCTAAATAGATTAAATGTGCTTTAACGAAGA

Luciferase

5' GGTGGCGCTCCCCTCTCTAAGGAAGTCGGGGAAGCGGTTGCCAAGAGGTTCCATCTGCCAGGTATCAGGCAAGGATATGGGCTCACTGAGACTACATCAGCTATTCTGAT 2200
3' CCACCGCGAGGGGAGAGATTCCTTCAGCCCCTTCGCCAACGGTTCTCCAAGGTAGACGGTCCATAGTCCGTTCCTATACCCGAGTGACTCTGATGTAGTCGATAAGACTA

Luciferase

5' TACACCCGAGGGGGATGATAAACCGGGCGCGGTCGGTAAAGTTGTTCCATTTTTTGAAGCGAAGGTTGTGGATCTGGATACCGGGAAAACGCTGGGCGTTAATCAAAGAG 2310
3' ATGTGGGCTCCCCCTACTATTTGGCCCGCGCCAGCCATTTCAACAAGGTAAAAAACTTCGCTTCCAACACCTAGACCTATGGCCCTTTTGCGACCCGCAATTAGTTTCTC

Luciferase

5' GCGAACTGTGTGTGAGAGGTCCTATGATTATGTCCGGTTATGTAAACAATCCGGAAGCGACCAACGCCTTGATTGACAAGGATGGATGGCTACATTCTGGAGACATAGCT 2420
3' CGCTTGACACACACTCTCCAGGATACTAATACAGGCCAATACATTTGTTAGGCCTTCGCTGGTTGCGGAACTAACTGTTCCTACCTACCGATGTAAGACCTCTGTATCGA

Luciferase

5' TACTGGGACGAAGACGAACACTTCTTCATCGTTGACCGCCTGAAGTCTCTGATTAAGTACAAAGGCTATCAGGTGGCTCCCGCTGAATTGGAATCCATCTTGCTCCAACA 2530
3' ATGACCCTGCTTCTGCTTGTGAAGAAGTAGCAACTGGCGGACTTCAGAGACTAATTCATGTTTCCGATAGTCCACCGAGGGCGACTTAACCTTAGGTAGAACGAGGTTGT

Luciferase

SgrAI

5' CCCCAACATCTTCGACGCAGGTGTCGCAGGTCTTCCCGACGATGACGCCGGTGAACTTCCCGCCGCCGTTGTTGTTTTGGAGCACGGAAAGACGATGACGGAAAAAGAGA 2640
3' GGGGTTGTAGAAGCTGCGTCCACAGCGTCCAGAAGGGCTGCTACTGCGGCCACTTGAAGGGCGGCGGCAACAACAAAACCTCGTGCCTTTCTGCTACTGCCTTTTTCTCT

Luciferase

5' TCGTGGATTACGTCGCCAGTCAAGTAACAACCGCGAAAAAGTTGCGCGGAGGAGTTGTGTTTGTGGACGAAGTACCGAAAGGTCTTACCGGAAAACTCGACGCAAGAAAA 2750
3' AGCACCTAATGCAGCGGTCAGTTCATTGTTGGCGCTTTTTCAACGCGCCTCCTCAACACAAACACCTGCTTCATGGCTTTCCAGAATGGCCTTTTGAGCTGCGTTCTTTT

Luciferase

XbaI FseI

5' ATCAGAGAGATCCTCATAAAGGCCAAGAAGGGCGGAAAGATCGCCGTGTAATTCTAGAGTCGGGGCGGCCGGCCGCTTCGAGCAGACATGATAAGATACATTGATGAGTT 2860
3' TAGTCTCTCTAGGAGTATTTCCGGTTCTTCCCGCCTTTCTAGCGGCACATTAAGATCTCAGCCCCGCCGGCCGGCGAAGCTCGTCTGTACTATTCTATGTAACTACTCAA

Luciferase SV40 late poly

HpaI

5' TGGACAAACCACAACTAGAATGCAGTGAAAAAAATGCTTTATTTGTGAAATTTGTGATGCTATTGCTTTATTTGTAACCATTATAAGCTGCAATAAACAAGTTAACAACA 2970
3' ACCTGTTTGGTGTTGATCTTACGTCACTTTTTTTACGAAATAAACACTTTAAACACTACGATAACGAAATAAACATTGGTAATATTCGACGTTATTTGTTCAATTGTTGT

SV40 late poly

BsaBI SalI
BamHI AccI

5' ACAATTGCATTCATTTTATGTTTCAGGTTCAGGGGGAGGTGTGGGAGGTTTTTTAAAGCAAGTAAAACCTCTACAAATGTGGTAAAATCGATAAGGATCCGTCGACCGAT 3080
3' TGTTAACGTAAGTAAAATACAAAGTCCAAGTCCCCCTCCACACCCTCCAAAAAATTTCGTTCATTTTGGAGATGTTTACACCATTTTAGCTATTCCTAGGCAGCTGGCTA

SV40 late poly

PshAI

5' GCCCTTGAGAGCCTTCAACCCAGTCAGCTCCTTCCGGTGGGCGCGGGGCATGACTATCGTCGCCGCACTTATGACTGTCTTCTTTATCATGCAACTCGTAGGACAGGTGC 3190
3' CGGGAACTCTCGGAAGTTGGGTCAGTCGAGGAAGGCCACCCGCGCCCCGTACTGATAGCAGCGGCGTGAATACTGACAGAAGAAATAGTACGTTGAGCATCCTGTCCACG

RVprimer4 binding site

AfeI

5' CGGCAGCGCTCTTCCGCTTCCTCGCTCACTGACTCGCTGCGCTCGGTCGTTCGGCTGCGGCGAGCGGTATCAGCTCACTCAAAGGCGGTAATACGGTTATCCACAGAATC 3300
3' GCCGTCGCGAGAAGGCGAAGGAGCGAGTGACTGAGCGACGCGAGCCAGCAAGCCGACGCCGCTCGCCATAGTCGAGTGAGTTTCCGCCATTATGCCAATAGGTGTCTTAG

PciI

5' AGGGGATAACGCAGGAAAGAACATGTGAGCAAAAGGCCAGCAAAAGGCCAGGAACCGTAAAAAGGCCGCGTTGCTGGCGTTTTTCCATAGGCTCCGCCCCCCTGACGAGC 3410
3' TCCCCTATTGCGTCCTTTCTTGTACACTCGTTTTCCGGTCGTTTTCCGGTCCTTGGCATTTTTCCGGCGCAACGACCGCAAAAAGGTATCCGAGGCGGGGGGACTGCTCG

5' ATCACAAAAATCGACGCTCAAGTCAGAGGTGGCGAAACCCGACAGGACTATAAAGATACCAGGCGTTTCCCCCTGGAAGCTCCCTCGTGCGCTCTCCTGTTCCGACCCTG 3520
3' TAGTGTTTTTAGCTGCGAGTTCAGTCTCCACCGCTTTGGGCTGTCCTGATATTTCTATGGTCCGCAAAGGGGGACCTTCGAGGGAGCACGCGAGAGGACAAGGCTGGGAC

5' CCGCTTACCGGATACCTGTCCGCCTTTCTCCCTTCGGGAAGCGTGGCGCTTTCTCATAGCTCACGCTGTAGGTATCTCAGTTCGGTGTAGGTCGTTCGCTCCAAGCTGGG 3630
3' GGCGAATGGCCTATGGACAGGCGGAAAGAGGGAAGCCCTTCGCACCGCGAAAGAGTATCGAGTGCGACATCCATAGAGTCAAGCCACATCCAGCAAGCGAGGTTCGACCC

5' CTGTGTGCACGAACCCCCCGTTCAGCCCGACCGCTGCGCCTTATCCGGTAACTATCGTCTTGAGTCCAACCCGGTAAGACACGACTTATCGCCACTGGCAGCAGCCACTG 3740
3' GACACACGTGCTTGGGGGGCAAGTCGGGCTGGCGACGCGGAATAGGCCATTGATAGCAGAACTCAGGTTGGGCCATTCTGTGCTGAATAGCGGTGACCGTCGTCGGTGAC

5' GTAACAGGATTAGCAGAGCGAGGTATGTAGGCGGTGCTACAGAGTTCTTGAAGTGGTGGCCTAACTACGGCTACACTAGAAGAACAGTATTTGGTATCTGCGCTCTGCTG 3850
3' CATTGTCCTAATCGTCTCGCTCCATACATCCGCCACGATGTCTCAAGAACTTCACCACCGGATTGATGCCGATGTGATCTTCTTGTCATAAACCATAGACGCGAGACGAC

5' AAGCCAGTTACCTTCGGAAAAAGAGTTGGTAGCTCTTGATCCGGCAAACAAACCACCGCTGGTAGCGGTGGTTTTTTTGTTTGCAAGCAGCAGATTACGCGCAGAAAAAA 3960
3' TTCGGTCAATGGAAGCCTTTTTCTCAACCATCGAGAACTAGGCCGTTTGTTTGGTGGCGACCATCGCCACCAAAAAAACAAACGTTCGTCGTCTAATGCGCGTCTTTTTT

5' AGGATCTCAAGAAGATCCTTTGATCTTTTCTACGGGGTCTGACGCTCAGTGGAACGAAAACTCACGTTAAGGGATTTTGGTCATGAGATTATCAAAAAGGATCTTCACCT 4070
3' TCCTAGAGTTCTTCTAGGAAACTAGAAAAGATGCCCCAGACTGCGAGTCACCTTGCTTTTGAGTGCAATTCCCTAAAACCAGTACTCTAATAGTTTTTCCTAGAAGTGGA

5' AGATCCTTTTAAATTAAAAATGAAGTTTTAAATCAATCTAAAGTATATATGAGTAAACTTGGTCTGACAGTTACCAATGCTTAATCAGTGAGGCACCTATCTCAGCGATC 4180
3' TCTAGGAAAATTTAATTTTTACTTCAAAATTTAGTTAGATTTCATATATACTCATTTGAACCAGACTGTCAATGGTTACGAATTAGTCACTCCGTGGATAGAGTCGCTAG

beta-lactamase

AhdI BsaI

5' TGTCTATTTCGTTCATCCATAGTTGCCTGACTCCCCGTCGTGTAGATAACTACGATACGGGAGGGCTTACCATCTGGCCCCAGTGCTGCAATGATACCGCGAGACCCACG 4290
3' ACAGATAAAGCAAGTAGGTATCAACGGACTGAGGGGCAGCACATCTATTGATGCTATGCCCTCCCGAATGGTAGACCGGGGTCACGACGTTACTATGGCGCTCTGGGTGC

beta-lactamase

```
                                                                                    NmeAIII                    AseI
5' CTCACCGGCTCCAGATTTATCAGCAATAAACCAGCCAGCCGGAAGGGCCGAGCGCAGAAGTGGTCCTGCAACTTTATCCGCCTCCATCCAGTCTATTAATTGTTGCCGGG  4400
3' GAGTGGCCGAGGTCTAAATAGTCGTTATTTGGTCGGTCGGCCTTCCCGGCTCGCGTCTTCACCAGGACGTTGAAATAGGCGGAGGTAGGTCAGATAATTAACAACGGCCC
   beta-lactamase

5' AAGCTAGAGTAAGTAGTTCGCCAGTTAATAGTTTGCGCAACGTTGTTGCCATTGCTACAGGCATCGTGGTGTCACGCTCGTCGTTTGGTATGGCTTCATTCAGCTCCGGT  4510
3' TTCGATCTCATTCATCAAGCGGTCAATTATCAAACGCGTTGCAACAACGGTAACGATGTCCGTAGCACCACAGTGCGAGCAGCAAACCATACCGAAGTAAGTCGAGGCCA
   beta-lactamase

5' TCCCAACGATCAAGGCGAGTTACATGATCCCCCATGTTGTGCAAAAAAGCGGTTAGCTCCTTCGGTCCTCCGATCGTTGTCAGAAGTAAGTTGGCCGCAGTGTTATCACT  4620
3' AGGGTTGCTAGTTCCGCTCAATGTACTAGGGGGTACAACACGTTTTTTCGCCAATCGAGGAAGCCAGGAGGCTAGCAACAGTCTTCATTCAACCGGCGTCACAATAGTGA
   beta-lactamase

5' CATGGTTATGGCAGCACTGCATAATTCTCTTACTGTCATGCCATCCGTAAGATGCTTTTCTGTGACTGGTGAGTACTCAACCAAGTCATTCTGAGAATAGTGTATGCGGC  4730
3' GTACCAATACCGTCGTGACGTATTAAGAGAATGACAGTACGGTAGGCATTCTACGAAAAGACACTGACCACTCATGAGTTGGTTCAGTAAGACTCTTATCACATACGCCG
   beta-lactamase

5' GACCGAGTTGCTCTTGCCCGGCGTCAATACGGGATAATACCGCGCCACATAGCAGAACTTTAAAAGTGCTCATCATTGGAAAACGTTCTTCGGGGCGAAAACTCTCAAGG  4840
3' CTGGCTCAACGAGAACGGGCCGCAGTTATGCCCTATTATGGCGCGGTGTATCGTCTTGAAATTTTCACGAGTAGTAACCTTTTGCAAGAAGCCCCGCTTTTGAGAGTTCC
   beta-lactamase

5' ATCTTACCGCTGTTGAGATCCAGTTCGATGTAACCCACTCGTGCACCCAACTGATCTTCAGCATCTTTTACTTTCACCAGCGTTTCTGGGTGAGCAAAAACAGGAAGGCA  4950
3' TAGAATGGCGACAACTCTAGGTCAAGCTACATTGGGTGAGCACGTGGGTTGACTAGAAGTCGTAGAAAATGAAAGTGGTCGCAAAGACCCACTCGTTTTTGTCCTTCCGT
   beta-lactamase

5' AAATGCCGCAAAAAAGGGAATAAGGGCGACACGGAAATGTTGAATACTCATACTCTTCCTTTTTCAATATTATTGAAGCATTTATCAGGGTTATTGTCTCATGAGCGGAT  5060
3' TTTACGGCGTTTTTTCCCTTATTCCCGCTGTGCCTTTACAACTTATGAGTATGAGAAGGAAAAAGTTATAATAACTTCGTAAATAGTCCCAATAACAGAGTACTCGCCTA
   beta-lactamase

5' ACATATTTGAATGTATTTAGAAAAATAAACAAATAGGGGTTCCGCGCACATTTCCCCGAAAAGTGCCACCTGACGCGCCCTGTAGCGGCGCATTAAGCGCGGCGGGTGTG  5170
3' TGTATAAACTTACATAAATCTTTTTATTTGTTTATCCCCAAGGCGCGTGTAAAGGGGCTTTTCACGGTGGACTGCGCGGGACATCGCCGCGTAATTCGCGCCGCCCACAC
                                                                                       f1 origin

5' GTGGTTACGCGCAGCGTGACCGCTACACTTGCCAGCGCCCTAGCGCCCGCTCCTTTCGCTTTCTTCCCTTCCTTTCTCGCCACGTTCGCCGGCTTTCCCCGTCAAGCTCT  5280
3' CACCAATGCGCGTCGCACTGGCGATGTGAACGGTCGCGGGATCGCGGGCGAGGAAAGCGAAAGAAGGGAAGGAAAGAGCGGTGCAAGCGGCCGAAAGGGGCAGTTCGAGA
   f1 origin

                                                                                              BsaAI
                                                                                         BtgZI    DraIII
5' AAATCGGGGGCTCCCTTTAGGGTTCCGATTTAGTGCTTTACGGCACCTCGACCCCAAAAAACTTGATTAGGGTGATGGTTCACGTAGTGGGCCATCGCCCTGATAGACGG  5390
3' TTTAGCCCCCGAGGGAAATCCCAAGGCTAAATCACGAAATGCCGTGGAGCTGGGGTTTTTTGAACTAATCCCACTACCAAGTGCATCACCCGGTAGCGGGACTATCTGCC
   f1 origin

5' TTTTTCGCCCTTTGACGTTGGAGTCCACGTTCTTTAATAGTGGACTCTTGTTCCAAACTGGAACAACACTCAACCCTATCTCGGTCTATTCTTTTGATTTATAAGGGATT  5500
3' AAAAAGCGGGAAACTGCAACCTCAGGTGCAAGAAATTATCACCTGAGAACAAGGTTTGACCTTGTTGTGAGTTGGGATAGAGCCAGATAAGAAAACTAAATATTCCCTAA
   f1 origin
```

5' TTGCCGATTTCGGCCTATTGGTTAAAAAATGAGCTGATTTAACAAAAATTTAACGCGAATTTTAACAAAATATTAACGCTTACAATTTGCCATTCGCCATTCAGGCTGCG 5610
3' AACGGCTAAAGCCGGATAACCAATTTTTTACTCGACTAAATTGTTTTTAAATTGCGCTTAAAATTGTTTTATAATTGCGAATGTTAAACGGTAAGCGGTAAGTCCGACGC

f1 origin

NotI

5' CAACTGTTGGGAAGGGCGATCGGTGCGGGCCTCTTCGCTATTACGCCAGCCCAAGCTACCATGATAAGTAAGTAATATTAAGGTACGGGAGGTACTTGGAGCGGCCGCAA 5720
3' GTTGACAACCCTTCCCGCTAGCCACGCCCGGAGAAGCGATAATGCGGTCGGGTTCGATGGTACTATTCATTCATTATAATTCCATGCCCTCCATGAACCTCGCCGGCGTT

5' TAAAATATCTTTATTTTCATTACATCTGTGTGTTGGTTTTTTGTGTGAATCGATAGTACTAACATACGCTCTCCATCAAAACAAAACGAAACAAAACAAACTAGCAAAAT 5830
3' ATTTTATAGAAATAAAAGTAATGTAGACACACAACCAAAAAACACACTTAGCTATCATGATTGTATGCGAGAGGTAGTTTTGTTTTGCTTTGTTTTGTTTGATCGTTTTA

Synthetic poly

RVprim... site

pGL3 Basic forward primer

5' AGGCTGTCCCCAGTGCAAGTGCAGGTGCCAGAACATTTCTCTATCGATA 5879
3' TCCGACAGGGGTCACGTTCACGTCCACGGTCTTGTAAAGAGATAGCTAT

Synthetic poly

RVprim... site
